# Vision-related quality of life considering both eyes: results from the German population-based Gutenberg Health Study (GHS)

**DOI:** 10.1186/s12955-019-1158-1

**Published:** 2019-06-06

**Authors:** Stefan Nickels, Alexander K. Schuster, Heike Elflein, Christian Wolfram, Andreas Schulz, Thomas Münzel, Manfred E. Beutel, Irene Schmidtmann, Robert P. Finger, Norbert Pfeiffer

**Affiliations:** 1 Department of Ophthalmology, University Medical Center of the Johannes Gutenberg-University Mainz, Langenbeckstr. 1, 55131 Mainz, Germany; 2 Preventive Cardiology and Preventive Medicine, Center for Cardiology, Cardiology I, University Medical Center of the Johannes Gutenberg-University Mainz, Mainz, Germany; 3 Center for Cardiology, Cardiology I, University Medical Center of the Johannes Gutenberg-University Mainz, Mainz, Germany; 4 Department of Psychosomatic Medicine and Psychotherapy, University Medical Center of the Johannes Gutenberg-University Mainz, Mainz, Germany; 5 Institute for Medical Biostatistics, Epidemiology and Informatics, University Medical Center of the Johannes Gutenberg-University Mainz, Mainz, Germany; 6 Department of Ophthalmology, University of Bonn, Bonn, Germany

## Abstract

**Purpose:**

Most definitions of visual impairment focus on the status of the better-seeing eye only, but this approach might underestimate the influence of the worse-seeing eye on the vision-related quality of life (VRQoL).

**Methods:**

We assessed distance-corrected visual acuity in both eyes and VRQoL using the “National Eye Institute 25-Item Visual Function Questionnaire” (NEI VFQ-25) in the German population-based Gutenberg Health Study. We calculated the Rasch-based visual functioning scale (VFS) and socioemotional scale (SES). We categorized the visual acuity of the better-seeing eye (BE) and worse-seeing eye (WE) as follows: (1) no visual impairment (VI) (< 0.32 logMAR)), (2) mild VI (0.32–0.5 logMAR), and (3) moderate to severe VI (> 0.5 logMAR). Next, the subjects were categorized as follows: both eyes with no VI (no/no), the better-seeing eye with no VI and the worse-seeing eye with mild VI (no/mild), no VI/severe VI (no/severe), both eyes with mild VI (mild/mild), light VI/severe VI (mild/severe), and both eyes with severe VI (severe/severe). We calculated the median scores for VFS and SES. We used linear regression to estimate the combined influence of BE/WE on VFS and SES.

**Results:**

We included 11,941 participants (49.9% female, age range: 35–74 years) with information on VRQoL and visual acuity. The median VFS/SES scores were 90/100 (no/no VI group), 84/97 (no/mild group), 81/94 (no/severe group), 70/90 (mild/mild group), 67/74 (mild/severe group), and 63/76 (severe/severe group). These differences were supported by the regression analysis results.

**Conclusion:**

Relying on the function of the better-seeing eye considerably underestimates the impact of visual impairment on VRQoL.

**Electronic supplementary material:**

The online version of this article (10.1186/s12955-019-1158-1) contains supplementary material, which is available to authorized users.

## Background

The assessment of visual impairment poses the analytical challenge that both eyes could be affected differently. Most common definitions of visual impairment meet this challenge by focusing on the visual function of the better-seeing eye only —e.g., the definition of blindness of the World Health Organization and Global Burden of Disease Study [1, 2]. However, there is increasing evidence that this approach underestimates the influence of the worse-seeing fellow eye on visual function and vision-related quality of life (VRQoL) [3, 4]. The Melbourne Visual Impairment Project showed that even unilateral vision loss caused difficulties in reading and recognizing faces and increased the risk of falling when away and becoming dependent in persons aged 40 years and older [5]. Additionally, there is evidence from outcome studies that patients who underwent cataract surgery in both eyes reported greater improvement in quality of life than patients who underwent surgery in only one eye [6, 7]. Studies in patients with age-related macular degeneration showed that bilateral manifestation is associated with lower VRQoL than unilateral manifestation [8]. Finger et al. showed that calculating patient reported utilizes based on the better-seeing eye only is likely to underestimate the impact of visual impairment [4]. To further elucidate the contribution of both eyes to VRQoL, we explored in a large population-based sample whether VRQoL is different when stratifying participants according to the visual acuity of their better-seeing eye only and compared this to also considering the visual acuity of the fellow eye (e.g., the worse-seeing eye). We hypothesize that there will be a substantial impact of the worse-seeing eye on VRQoL.

## Materials and methods

### Study population

We analyzed a subsample of the Gutenberg Health Study (GHS) with data on both visual acuity and VRQoL available. The GHS is a population-based, single-center, prospective, cohort study at the medical center of the Johannes Gutenberg University Mainz in Germany [9]. The population sample was randomly drawn via local residents’ registration offices and equally stratified by sex for each decade of age. The baseline examination included 15,010 participants aged 35 to 74 years and was conducted from 2007 to 2012. The examination consisted of an ophthalmological examination, general and cardiovascular examinations, and questionnaires and interviews. The ophthalmic branch has been described in detail by Höhn et al. [10]. Briefly, we conducted measurements of autorefraction and distance-corrected visual acuity, intraocular pressure, visual field testing, pachy- and keratometry, and posterior segment photography. We used the NEI VFQ-25 to assess VRQoL. We included all participants who completed this questionnaire and with available information on the visual acuity of both eyes.

### VRQoL data acquisition and analysis

Vision-related QoL was assessed using the German version of the NEI VFQ-25 [11, 12]. The German NEI VFQ-25 has been assessed for its psychometric properties and was used by various studies [12–17]. The questionnaire was self-administered as a print-out at the study site and completed using both eyes and reading glasses, if necessary. We used the most common VRQoL instrument and applied state-of-the-art techniques remediating the known flaws—i.e., the violation of unidimensionality and lack of interval-scaled measurements—to analyze the NEI VFQ-25 data. We followed the suggestions of Pesudovs to calculate the “visual functioning scale (VFS)” and “socioemotional scale” (SES); both are interval scaled (0 = worst to 100 = best) and based on the Rasch-transformed individual-level NEI VFQ-25 data [18, 19]. The developers of the questionnaire initially proposed to calculate 12 subscores and one composite VRQoL score (http://www.rand.org/health/surveys_tools/vfq.html, last accessed 2018-03-27) [18]. Subsequent studies showed that this approach violates unidimensionality and interval-level measurement, both important properties of an instrument measuring QoL [20–24]. Therefore, we chose the Rasch-based approach described above, as previously used by us and others [18, 19, 25, 26].

### Ocular measurements

Objective refraction and distance-corrected visual acuity were measured in both eyes using a Humphrey Automated Refractor/Keratometer (HARK) 599 (Carl Zeiss AG, Jena, Germany) without cycloplegia, starting with the right eye [10]. Distance-corrected visual acuity was measured using the built-in Snellen charts, ranging from 20/400 to 40/20 (logMAR 1.3 to − 0.3). Below that visual acuity, we used a visual acuity chart at a distance of one meter up to 20/800 (logMAR 1.6), followed by counting fingers, hand movements, and the light perception test. The spherical equivalent was calculated as the spherical correction value plus half of the cylindrical power. A history of eye disease was assessed in a short interview preceding the eye examination.

### Sociodemographic characteristics

The socioeconomic status was based on income, education and occupation and was defined according to the German Health Update 2009 (GEDA), with a range from 3 (lowest) to 21 (highest) socioeconomic status [27].

### Statistical analysis

For descriptive analyses, we calculated the mean of the spherical equivalent of both eyes for every participant. Visual acuity measurements were converted to logMAR [28]. We categorized the visual acuity of the better-seeing eye and worse-seeing eye as follows, with the thresholds based on the WHO definition of visual impairment (VI): (0) no VI (< 0.32 logMAR)), (1) mild VI (0.32–0.5 logMAR), and (2) moderate to severe VI (> 0.5 logMAR) [1, 4]. The subjects were then categorized in 6 groups according to the VI categories of both eyes (Table 1).

**Table 1:** Cross-table illustrating the categorization of subjects according to visual impairment of both eyes in the population-based Gutenberg Health Study (2007–2012)

		Worse-seeing eye:		
		No VI	Mild VI	Moderate/severe
Better-seeing eye:	No VI	“b0/w0”, *n* = 11,021	“b0/w1”, *n* = 430	“b0/w2”, *n* = 395
	Mild VI	/	“b1/w1”, *n* = 35	“b1/w2”, *n* = 34
	Moderate/severe VI	/	/	“b2/w2”, *n* = 26

*BE* Better-seeing eye, *WE* Worse-seeing eye, *VI* Vision impairment; no VI: < 0.32 logMAR, mild VI: 0.32–0.5 logMAR; moderate/severe VI: > 0.5 logMAR

We calculated the median and interquartile range for both VFS and SES for each group. We used linear regression models to estimate the influence of the combination of BE/WE on the VRQoL scores (reference group: no visual impairment in both eyes). We adjusted for age and sex in the basic model and additionally for socioeconomic status in a second model. Additionally, we calculated separate models for participants aged younger than 65 years and those aged 65 years and older. We repeated the analyses restricted to participants without self-reported amblyopia. Amblyopia is characterized by reduced vision since childhood and might have a different impact on VRQoL than visual impairment acquired in later life as affected persons are used to having “one lazy eye” (i.e., only one functioning eye) [29]. The VRQoL might additionally be affected by other nonocular chronic diseases. Therefore, we also calculated models restricted to participants without the following chronic diseases: diabetes mellitus, cardiovascular disease, peripheral artery disease, chronic kidney disease, chronic liver disease, chronic obstructive pulmonary disease, depression, and cancer. Due to the exploratory character of this analysis, *p*-values were not adjusted for multiple testing. The data were analyzed using GNU R version 3.3.1 [30].

## Results

Our sample consisted of 11,941 GHS participants (49.9% female; median age: 54.7 years) who completed the questionnaire without missing items necessary to calculate the VRQoL scales and with visual acuity of both eyes available. Categorized by the better-seeing eye, *n* = 11,846 (99.2%) had no VI, *n* = 69 (0.6%) had mild VI, and *n* = 26 (0.2%) had moderate to severe VI. The median VRQoL score of the visual functioning scale (VFS) was 89.6 and that of the socioemotional scale (SES) was 100.0. Details of the study sample are shown in Table 2. Characteristics stratified by sex are shown in the (Additional file 1: Table S1).

**Table 2:** Characteristics of the study sample of the German population-based Gutenberg Health Study (GHS) with visual acuity of both eyes and NEI VFQ-25 data available

	All
N	11,941
Age [y]	54.7 (11.1)
Women	49.9% (5958)
Socioeconomic status	13.1 (4.4)
Eye characteristics:	
Mean spherical equivalent [dpt]	−0.12 (−1.25/0.81)
Visual acuity (better eye) [logMAR]	0 (0/0.10)
Visual acuity (worse eye) [logMAR]	0 (0/0.22)
Contact lenses or glasses	89.2% (10646)
Distance glasses	68.0% (8116)
History of eye surgery	7.3% (876)
Glaucoma	2.3% (270)
Age-related macular degeneration	0.4% (51)
Amblyopia	9.9% (1186)
Vision-related quality of life:	
Visual functioning scale (NEI VFQ-25)	89.6 (81.3/95.1)
Socioemotional scale (NEI VFQ-25)	100.0 (94.5/100.0)

Continuous variables were described by mean values and standard deviation, and a skewed distribution was described by the median and interquartile range. Discrete variables were described by relative and absolute frequencies.

**Fig. 1:**
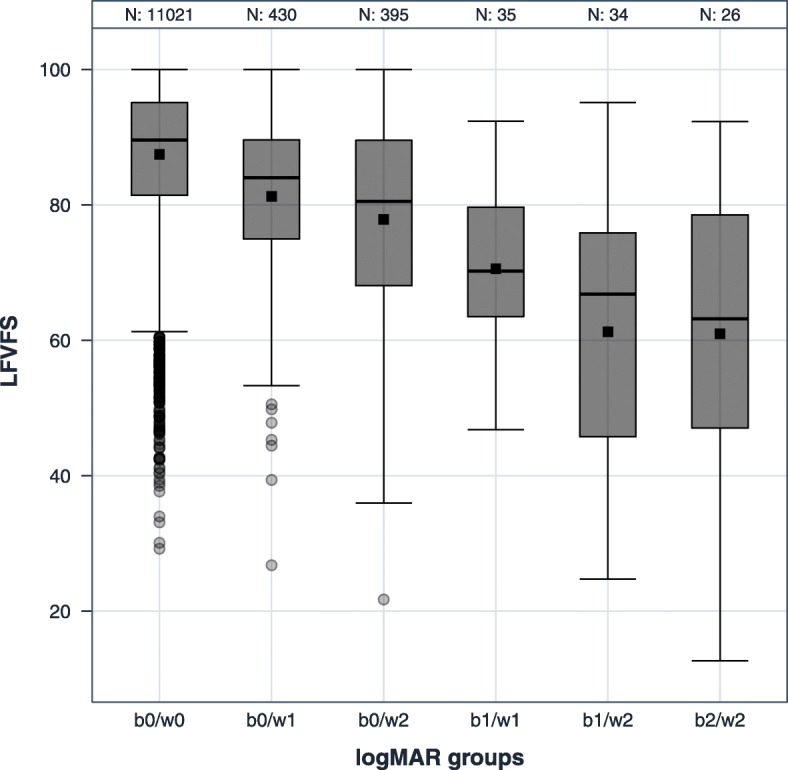
Distribution of NEI VFQ-25 visual functioning scale scores by categories of vision impairment (by the better-seeing eye and worse-seeing eye) of the German population-based Gutenberg Health Study (GHS), 2007–2012. b: better-seeing eye; w: worse-seeing eye; 0: no visual impairment (< 0.32 logMAR), 1: mild vision impairment (0.32–0.5 logMAR); 2: moderate/severe vision impairment (> 0.5 logMAR); gray boxes: interquartile range (IQR); black line: median; black square: mean; whiskers: lowest data point still within 1.5 (IQR) of the lower quartile, and the highest data point still within 1.5 IQR of the upper quartile; dots: outliers

Categorized only by the better eye, the median scores for the VFS were 89.6 (no VI), 70.2 (mild VI), and 63.2 (moderate to severe VI) (Table 3). If the VI of the worse-seeing eye was considered additionally, there was a decrease of approximately four points per VI category in the strata defined by the better-seeing eye (Tables 4 and 5, Fig 1). Participants with no visual impairment in the better-seeing eye and mild visual impairment in the fellow eye had a 5.0-point lower VFS score than participants with no visual impairment in both eyes (95% confidence interval (CI) -6.0; − 4.1). Participants with no visual impairment in the better-seeing eye and severe visual impairment in the fellow eye had a 9.0-point lower VFS score than participants with no visual impairment in both eyes (95% CI: − 10.0; − 8.0). These estimates are from the regression model adjusted for age, sex and socioeconomic status (Table 6). Different adjustment sets or restrictions to a subsample without general comorbidities or amblyopia resulted in only minor changes in the estimates (Additional files 2, 3, 4, 5, 6 and 7). For all regression coefficients in the main analyses, p was below 0.0001.

**Table 3:** NEI VFQ-25 visual functioning scale scores and socioemotional scale scores by categories of vision impairment of the better-seeing eye only in the German population-based Gutenberg Health Study (GHS), 2007–2012

Visual impairment of the better-seeing eye	visual functioning scale score (VFS)	socioemotional scale score (SES)
No VI (*n* = 11,846)	89.6 (81.3/95.1)	100 (94.6/100)
Mild VI (*n* = 69)	70.2 (57.6/78.0)	88.4 (67.9/97.1)
Moderate/severe VI (*n* = 26)	63.2 (46.6/78.7)	76.3 (68.0/93.9)

Scores displayed as medians (interquartile range); *VI* Vision impairment; no VI: < 0.32 logMAR, mild VI: 0.32–0.5 logMAR; moderate/severe VI: > 0.5 logMAR

**Table 4:** NEI VFQ-25 visual functioning scale scores by categories of vision impairment considering both the better-seeing eye and worse-seeing eye of the German population-based Gutenberg Health Study (GHS), 2007–2012

		Worse-seeing eye		
		No VI (*n* = 11,021)	Mild VI (*n* = 465)	Moderate/severe VI (*n* = 455)
Better-seeing eye	No VI	89.6 (81.4/95.1), *n* = 11,021	84.0 (74.9/89.6), *n* = 430	80.5 (68.0/89.5), *n* = 395
	Mild VI	/	70.2 (62.5/80.2), *n* = 35	66.8 (45.7/76.0), *n* = 34
	Moderate/severe VI	/	/	63.2 (46.6/78.7), *n* = 26

Scores displayed as medians (interquartile range), *VI* Vision impairment; no VI: < 0.32 logMAR, mild VI: 0.32–0.5 logMAR; moderate/severe VI: > 0.5 logMAR

**Table 5:** NEI VFQ-25 socioemotional scale scores by categories of vision impairment (by the better-seeing eye and worse-seeing eye) of the German population-based Gutenberg Health Study (GHS), 2007–2012

		Worse-seeing eye		
		No VI (*n* = 11,021)	Mild VI (*n* = 465)	Moderate/severe VI (*n* = 455)
Better-seeing eye	No VI	100 (95.1/100), *n* = 11,021	97.1 (91.2/100), *n* = 430	94.1 (85.8/100), *n* = 395
	Mild VI	/	90.1 (72.4/97.1), *n* = 35	73.7 (67.5/95.2), *n* = 34
	Moderate/severe VI	/	/	76.3 (68.0/93.9), *n* = 26

Scores displayed as medians (interquartile range); *VI* Vision impairment; no VI: < 0.32 logMAR, mild VI: 0.32–0.5 logMAR; moderate/severe VI: > 0.5 logMAR)

**Table 6:** Linear regression estimates of the influence of both the better-seeing and worse-seeing eyes on the NEI VFQ-25 visual functioning scale score in the German population-based Gutenberg Health Study (GHS), 2007–2012

Category of visual impairment considering better-seeing and worse-seeing eye	Model 1a	Model 1b
	(*n* = 11,941, R^2^ = 0.12)	(*n* = 11,889, R^2^ = 0.12)
	Estimate (CI)	Estimate (CI)
BE no VI, WE mild VI	−5.2^a^	−5.0^a^
	(−6.1; −4.2)	(−6.0; −4.1)
BE no VI, WE moderate/ severe VI	−9.03^a^	−9.01^a^
	(−10.0; −8.0)	(−10.0; −8.0)
BE mild VI & WE mild VI	−14.3^a^	− 14.5^a^
	(−17.6; − 11.0)	(− 17.8; − 11.1)
BE mild VI & WE moderate/ severe VI	−24.3^a^	−24.1^a^
	(−27.7; − 21.0)	(− 27.4; -20.8)
BE moderate/ severe VI, WE moderate/ severe VI	− 25.3^a^	− 24.4^a^
	(−29.1; − 21.5)	(− 28.3; −20.5)

*BE* Better-seeing eye, *WE* Worse-seeing eye, *VI* Vision impairment; no VI: < 0.32 logMAR, mild VI: 0.32–0.5 logMAR; moderate/severe VI: > 0.5 logMAR); all models adjusted for age and sex using both eyes with no VI as a reference; model 1b additionally adjusted for socioeconomic status; CI: 95% confidence interval; ^a^: *p* < 0.0001, R^2^: adjusted R^2^

**Fig. 2:**
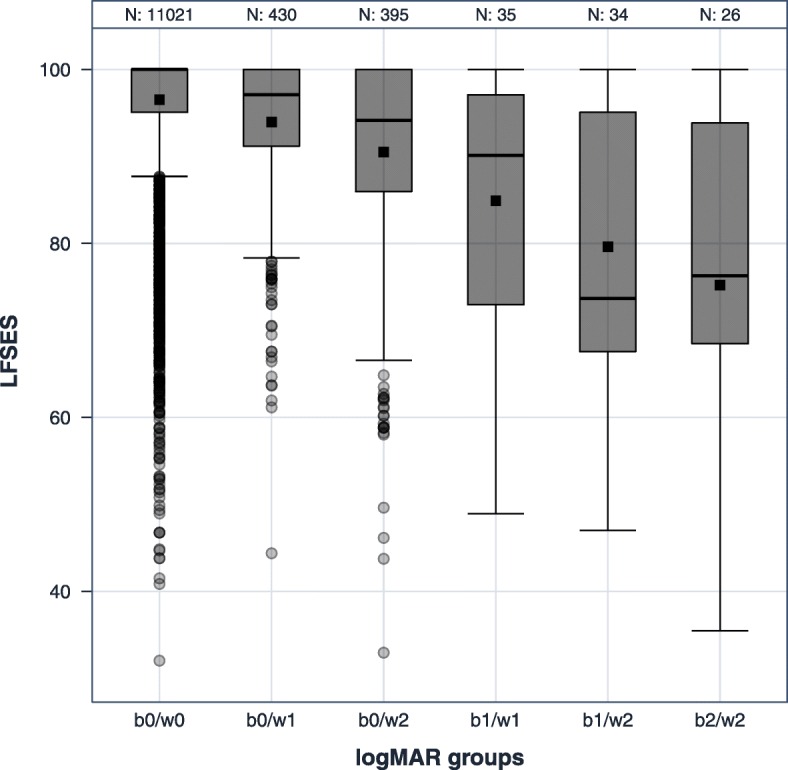
Distribution of NEI VFQ-25 socioemotional scale scores by categories of vision impairment (by better-seeing eye and worse-seeing eye) of the German population-based Gutenberg Health Study (GHS), 2007–2012. b: better-seeing eye; w: worse-seeing eye; 0: no vision impairment (< 0.32 logMAR), 1: mild vision impairment (0.32–0.5 logMAR); 2: moderate/severe vision impairment (> 0.5 logMAR); gray boxes: interquartile range (IQR); black line: median; black square: mean; whiskers: lowest data point still within 1.5 (IQR) of the lower quartile, and the highest data point still within 1.5 IQR of the upper quartile; dots: outliers

The median scores for the SES were 100 (no VI), 88.4 (mild VI), and 76.3 (moderate to severe VI) (Table 3). If the worse-seeing eye was considered additionally, there was a decrease of approximately three points per VI category in the stratum of no VI in the better-seeing eye (Tables 6 and 7, Fig. 2). In the stratum of the better-seeing eye with mild VI, there was a decrease of 16 points. Participants with no visual impairment in the better-seeing eye and mild visual impairment in the fellow eye had a 1.9-point lower SES score than participants with no visual impairment in both eyes. Participants with no visual impairment in the better-seeing eye and severe visual impairment in the fellow eye had a 5.7-point lower SES score than participants with no visual impairment in both eyes (95% CI -6.4; − 5.0). These estimates were from the regression model adjusted for age, sex and socioeconomic status (Table 7). The regression results also showed a substantial contribution of both eyes on SES. Adjustment for different potential confounders and excluding general comorbidities did not substantially change the regression estimates. In the subsample without amblyopia, the influence of a worse-seeing eye with moderate/severe VI in addition to a better-seeing eye with no VI was larger than that in the whole sample, and the effect size was similar to mild VI in both eyes (Additional files 2, 3, 4, 5, 6 and 7). For all regression coefficients in the main analyses, p was below 0.0001.

**Table 7:** Linear regression estimates of the influence of both the better-seeing and worse-seeing eyes on the NEI VFQ-25 socioemotional scale score in the German population-based Gutenberg Health Study (GHS), 2007–2012

Category of visual impairment considering better-seeing and worse-seeing eye	Model 1a	Model 1b
	(*n* = 11,941, R^2^ = 0.09)	(*n* = 11,889, R^2^ = 0.09)
	Estimate (CI)	Estimate (CI)
BE no VI, WE mild VI	−2.0^a^	− 1.9^a^
	(−2.6; −1.3)	(−2.6; − 1.2)
BE no VI, WE mild VI	−5.7^a^	− 5.7^a^
	(−6.4; − 5.0)	(− 6.4; − 5.0)
BE mild VI & WE mild VI	− 10.4^a^	−9.6^a^
	(−12.8; − 8.1)	(− 12.0; −7.2)
BE mild VI & WE moderate/ severe VI	− 16.0^a^	−15.9^a^
	(−18.4; −13.6)	(− 18.3; − 13.5)
BE moderate/ severe VI, WE moderate/ severe VI	−20.9^a^	−19.8^a^
	(−23.6; − 18.2)	(−22.6; − 17.1)

*BE* Better-seeing eye, *WE* Worse-seeing eye, *VI* Vision impairment; no VI: < 0.32 logMAR, mild VI: 0.32–0.5 logMAR; moderate/severe VI: > 0.5 logMAR; all models adjusted for age and sex using both eyes with no VI as reference; model 1b additionally adjusted for socioeconomic status; CI: 95% confidence interval; ^a^: *p* < 0.0001, R^2^: adjusted R^2^

In addition to adjusting for age, we calculated separate regression models for participants aged younger than 65 years and those aged 65 years and older. In the younger group, the impact of VI on VFS scores was higher; in both age groups, there was a substantial difference in VFS scores between categories defined by VI of both eyes. For the SES score, the decrease in the groups including the better-seeing eye without visual impairment was larger in the older participants. In the other VI groups, the reduction was more substantial in the younger age group (Additional files 2, 3, 4, 5, 6 and 7).

## Discussion

This study showed that the visual acuity of the worse-seeing eye has an important impact on VRQoL in addition to the impact of the better-seeing eye. In a large population-based setting, we showed the additional influence of the worse-seeing eye on VRQoL measured by the most commonly used VRQoL instrument, the NEI VFQ-25. The “one-eye approach”, although common, is very likely to neglect the patient’s perspective. Our results are in line with previous studies. Finger et al. used the same VI categories to evaluate the impact of the visual impairment of both eyes on patient-reported preferences but did use two other instruments instead of the NEI VFQ-25. There was no effect captured by generic EQ-5D, but the worse-seeing eye had an additional impact on the Vision and Quality of Life Index [4]. Varma et al. showed, in 5270 participants of the Los Angeles Latino Eye Study, that the impacts of unilateral moderate/severe VI and bilateral mild VI on VRQoL were similar, considering that only VI of one eye would have underestimated the real impact [31]. Hirneiss et al. reviewed the influence of both eyes on VRQoL in the context of treatment decisions based on 47 publications [3]. Despite the heterogeneity in study designs and methods applied to assess VRQoL, the authors conclude that treatment of both the better-seeing and worse-seeing eye leads to a benefit in the patients’ quality of life, contrary to the common assumption that the better-seeing eye mostly determines VRQoL [3]. There is evidence that, in age-related macular degeneration (AMD), VRQoL is lower in subjects with both eyes affected than in unilateral affected subjects. Dong et al. estimated a difference of six points between both groups when analyzing the NEI VFQ-25 scores [8]. Treatment of AMD leads to an improvement of VRQoL, as shown with the NEI VFQ-25 questionnaire, regardless of whether the better-seeing or worse-seeing eye was treated [32–34]. For cataract surgery, multiple studies reported an increase of VRQoL in subjects after cataract surgery of the second eye [6, 35, 36]. Schuster et al. used Rasch-transformed NEI VFQ-25 data from the GHS and reported that VRQoL has a similar magnitude in bilateral phakic and pseudophakic subjects, but monolateral pseudophakic subjects have a 6-point lower VRQoL [37]. These results underline the importance of considering both eyes when assessing the impact of VI on patient-reported outcomes.

There is no minimal clinically important difference (MCID) for Rasch-transformed NEI VFQ-25 scores; however, because the scale range is similar to the traditional scale ranges (both 0–100 points), the MCID magnitude is expected to be similar. The MCID for traditional NEI VFQ-25 scores is estimated to be four to six points [38, 39].

The strengths of our study are the large sample size combined with the population-based sampling. The broad assessment of phenotype information is based on a standardized study design and quality controls. Despite the population-based sampling, individuals with bilateral visual impairment are likely to be underrepresented in the GHS cohort because of their lower likelihood to participate. Furthermore, we had a considerable share of participants who did not complete the NEI VFQ-25. At the beginning of the GHS, participants were asked to complete the questionnaire at home. This was then changed to an on-site procedure that greatly increased the rate of participation. Participants with missing NEI VFQ-25 had a reduced physical health, but this seems to be mostly related to general health and less to self-reported eye diseases and ocular parameters [19]. Therefore, we assume the bias due to missing information on VRQoL to be small. Additionally, the numbers in the groups with advanced stages or VI are low, and most participants had no VI. This was to be expected, given the low prevalence of VI in European populations below the age of 75 years and in our population-based study sample [40]. This leads to a reduced precision of the estimates in the groups with advanced stages of VI. Nevertheless, we could show an additional impact on VRQoL of the worse-seeing eye compared with considering the better-seeing eye only. Because the GHS includes a recall every 5 years, we are planning to rerun the analyses in a 10-year older population in the future, where we expect a higher prevalence in VI and to see even more distinct results.

## Conclusion

In summary, we demonstrate in a large population-based study that the visual acuity of the worse-seeing eye has an important impact on VRQoL, in addition to the impact of the better-seeing eye. Whenever possible, the function of both eyes should be considered in medical decision making, as well as in clinical, health care and public health research.

## Additional files

Additional file 1: **Table S1.** Characteristics of the study sample of the German population-based Gutenberg Health Study (GHS) with the visual acuity of both eyes and NEI VFQ-25 data available, stratified by sex.. (PDF 219 kb)

Additional file 2: **Table S2a.** Linear regression estimates of the influence of both the better-seeing and worse-seeing eyes on the NEI VFQ-25 visual functioning scale score in the German population-based Gutenberg Health Study (GHS), 2007–2012, restricted to participants without chronic diseases or amblyopia. (PDF 218 kb)

Additional file 3: **Table S2b.** Linear regression estimates of the influence of both the better-seeing and worse-seeing eyes on the NEI VFQ-25 socioemotional scale score in the German population-based Gutenberg Health Study (GHS), 2007–2012, restricted to participants without chronic diseases or amblyopia. (PDF 218 kb)

Additional file 4: **Table S3a.** Linear regression estimates of the influence of both the better-seeing and worse-seeing eyes on the NEI VFQ-25 visual functioning scale score in the German population-based Gutenberg Health Study (GHS), 2007–2012, restricted to participants aged younger than 65 years. (PDF 215 kb)

Additional file 5: **Table S3b.** Linear regression estimates of the influence of both the better-seeing and worse-seeing eyes on the NEI VFQ-25 visual functioning scale score in the German population-based Gutenberg Health Study (GHS), 2007–2012, restricted to participants aged 65 years and older. (PDF 215 kb)

Additional file 6: **Table S4a.** Linear regression estimates of the influence of both the better-seeing and worse-seeing eye on the NEI VFQ-25 socioemotional scale score in the German population-based Gutenberg Health Study (GHS), 2007–2012, restricted to participants aged younger than 65 years. (PDF 215 kb)

Additional file 7: **Table S4b.** Linear regression estimates of the influence of both the better-seeing and worse-seeing eyes on the NEI VFQ-25 socioemotional scale score in the German population-based Gutenberg Health Study (GHS), 2007–2012, restricted to participants aged 65 years and older. (PDF 215 kb)

## Abbreviations

BSE: Better-seeing eye
CI: Confidence interval
GHS: Gutenberg health study
NEI VFQ-25: National Eye Institute 25-item visual function questionnaire
QoL: Quality of life
SES: Socioemotional scale
VFS: Visual functioning scale
VRQoL: Vision-related QoL
WSE: Worse-seeing eye
