# Supplementary material for: Vision-related quality of life considering both eyes: results from the German population-based Gutenberg Health Study (GHS)
Source: Health Qual Life Outcomes. 2019 Jun 6;17:98. doi: 10.1186/s12955-019-1158-1 (PMC6554962; doi:10.1186/s12955-019-1158-1)
Supplement: Supplementary file 1 — Table S1. Characteristics of the study sample of the German population-based Gutenberg Health Study (GHS) with the visual acuity of both eyes and NEI VFQ-25 data available, stratified by sex.. (PDF 219 kb) [file 12955_2019_1158_MOESM1_ESM.pdf]

Supplementary tables

Additional file 1: Table S1: Characteristics of the study sample of the German population-based Gutenberg Health Study (GHS) with visual acuity of both eyes and NEI VFQ-25 data available, stratified by sex.

	**Men**	**Women**
**n**	50.1% (5983)	49.9% (5958)
**Age [y]**	55.1 (11.1)	54.4 (11.1)
**Socio-economic status**	13.9 (4.5)	12.4 (4.1)
**Eye characteristics:**		
**Mean spherical equivalent [dpt]**	-0.12 (-1.25/0.75)	-0.06 (-1.19/0.88)
**Visual acuity (better eye) [logMAR]**	0 (0/0.10)	0 (0/0.10)
**Visual acuity (worse eye) [logMAR]**	0 (0/0.10)	0.10 (0/0.22)
**Contact lenses or glasses**	88.1% (5271)	90.2% (5375)
**Distance glasses**	66.7% (3989)	69.3% (4127)
**History of eye surgery**	6.9% (412)	7.8% (464)
**Glaucoma**	2.2% (133)	2.3% (137)
**Age-related macular degeneration**	0.4% (25)	0.4% (26)
**Amblyopia**	10.0% (600)	9.8% (586)
**Vision-related quality of life:**		
**Visual functioning scale (NEI VFQ-25)**	89.6 (81.5/95.1)	89.5 (78.8/94.5)
**Socioemotional scale (NEI VFQ-25)**	100.0 (94.6/100.0)	100.0 (94.2/100.0)
**Comorbidities:**		
**Cardiovascular disease**	12.1% (726)	5.3% (315)
**Peripheral artery disease**	3.2% (191)	2.9% (170)
**Diabetes mellitus**	10.8% (648)	6.1% (360)
**Chronic kidney disease**	1.2% (71)	1.0% (57)
**Chronic liver disease**	0.5% (32)	0.9% (51)
**Chronic obstructive pulmonary disease**	4.1% (246)	5.5% (329)
**Cancer**	8.2% (490)	9.9% (588)
**Depression**	6.0% (359)	9.2% (545)

Diabetes mellitus was defined by fulfilling one of the following criteria: diabetes mellitus diagnosed by a physician, known therapy (oral medication or insulin), or HbA1c >=6.5%. Depression was defined using the Patient Health Questionnaire (PHQ-9), using a sum score threshold of >=10. Cardiovascular disease: History of either myocardial infarction, coronary artery disease, atrial fibrillation, chronic heart failure; chronic obstructive pulmonary disease: history of asthma bronchiale or bronchitis; continuous variables are described by mean values and standard deviation or for skewed distribution by median and inter-quartile range. Discrete variables are described by relative and absolute frequencies.
