# Supplementary material for: Vision-related quality of life considering both eyes: results from the German population-based Gutenberg Health Study (GHS)
Source: Health Qual Life Outcomes. 2019 Jun 6;17:98. doi: 10.1186/s12955-019-1158-1 (PMC6554962; doi:10.1186/s12955-019-1158-1)
Supplement: Supplementary file 2 — Table S2a. Linear regression estimates of the influence of both the better-seeing and worse-seeing eyes on the NEI VFQ-25 visual functioning scale score in the German population-based Gutenberg Health Study (GHS), 2007–2012, restricted to participants without chronic diseases or amblyopia. (PDF 218 kb) [file 12955_2019_1158_MOESM2_ESM.pdf]

Additional file 2: Table S2: Linear regression estimates of the influence of both the better-seeing and the worse-seeing eye on the NEI VFQ-25 visual functioning scale score in the German population-based Gutenberg Health Study (GHS), 2007-2012, restricted to participants without chronic diseases or amblyopia.

**Category of visual impairment considering better-seeing and worse-seeing eye**	**Model S1 (n= 7964, R^2=0.11): Estimate (CI)**	**Model S2 (n= 7940, R^2=0.11): Estimate (CI)**	**Model S3 (n= 10753, R^2=0.12): Estimate (CI)**	**Model S4 (n= 10704, R^2=0.13): Estimate (CI)**
**BE no VI, WE mild VI**	-5.9* (-7.1; -4.8)	-5.8* (-7.0; -4.7)	-5.2* (-6.4; -3.9)	-5.0* (-6.3; -3.7)
**BE no VI, WE moderate/ severe VI**	-7.7* (-8.9; -6.6)	-7.7* (-8.9; -6.6)	-12.3* (-13.9; -10.7)	-12.3* (-13.9; -10.7)
**BE mild VI & WE mild VI**	-10.4* (-15.8; -5.1)	-11.9* (-17.5; -6.3)	-13.9* (-17.5; -10.4)	-14.2* (-17.9; -10.6)
**BE mild VI & WE moderate/ severe VI**	-23.5* (-28.2; -18.7)	-23.5* (-28.2; -18.7)	-21.1* (-25.2; -17.1)	-20.9* (-24.9; -16.8)
**BE moderate/ severe VI, WE moderate/ severe VI**	-22.2* (-27.5; -17.2)	-22.3* (-27.4; -17.1)	-33.7* (-39.2; -28.2)	-32.9* (-38.6; -27.2)

Model S1 restricted to participants without the following chronic diseases: diabetes mellitus, myocardial infarction, coronary artery disease, atrial fibrillation, chronic heart failure, peripheral artery disease, chronic kidney disease, chronic liver disease, asthma bronchiale, chronic bronchitis, depression, cancer; model S2 additionally adjusted for socio-economic status; model S3 restricted to participants without self-reported amblyopia, model S4 additionally adjusted for socio-economic status; CI: 95% confidence interval; *: $p<0.0001$, R^2: adjusted R^2.
