# Supplementary material for: Vision-related quality of life considering both eyes: results from the German population-based Gutenberg Health Study (GHS)
Source: Health Qual Life Outcomes. 2019 Jun 6;17:98. doi: 10.1186/s12955-019-1158-1 (PMC6554962; doi:10.1186/s12955-019-1158-1)
Supplement: Supplementary file 3 — Table S2b. Linear regression estimates of the influence of both the better-seeing and worse-seeing eyes on the NEI VFQ-25 socioemotional scale score in the German population-based Gutenberg Health Study (GHS), 2007–2012, restricted to participants without chronic diseases or amblyopia. (PDF 218 kb) [file 12955_2019_1158_MOESM3_ESM.pdf]

Additional file 3: Table S3: Linear regression estimates of the influence of both the better-seeing and the worse-seeing eye on the NEI VFQ-25 socio-emotional scale score in the German population-based Gutenberg Health Study (GHS), 2007-2012, restricted to participants without chronic diseases or amblyopia.

Category of visual impairment considering better-seeing and worse-seeing eye	Model S1 (n=7964, R^2=0.07): Estimate (CI)	Model S2 (n=7940, R^2=0.08): Estimate (CI)	Model S3 (n= 10753, R^2=0.09): Estimate (CI)	Model S4 (n= 10704, R^2=0.09): Estimate (CI)
**BE no VI, WE mild VI**	-2.2* (-2.9; -1.4)	-2.1* (-2.9; -1.4)	-2.84* (-3.75; -1.92)	-2.80* (-3.71; -1.90)
**BE no VI, WE mild VI**	-4.5* (-5.3; 3.8)	-4.5* (-5.3; -3.8)	-8.69* (-9.82; -7.56)	-8.77* (-9.90; -7.65)
**BE mild VI & WE mild VI**	-6.9* (-10.4; -3.3)	-8.0* (-11.7; -4.3)	-10.9* (-13.4; -8.42)	-10.1* (-12.6; -7.48)
**BE mild VI & WE moderate/ severe VI**	-16.4* (-19.5; -13.2)	-16.4* (-19.5, -13.2)	-17.3* (-20.2; -14.4)	-17.2* (-20.0; -14.3)
**BE moderate/ severe VI, WE moderate/ severe VI**	-17.0* (-20.4; -13.6)	-16.9* (-20.3; -13.5)	-26.7* (-30.6; -22.9)	-25.2* (-29.2; -21.2)

Model S1 restricted to participants without the following chronic diseases: diabetes mellitus, myocardial infarction, coronary artery disease, atrial fibrillation, chronic heart failure, peripheral artery disease, chronic kidney disease, chronic liver disease, asthma bronchiale, chronic bronchitis, depression, cancer; model S2 additionally adjusted for socio-economic status; model S3 restricted to participants without self-reported amblyopia, model S4 additionally adjusted for socio-economic status; CI: 95% confidence interval; *: $p<0.0001$, R^2: adjusted R^2.
