# Supplementary material for: Vision-related quality of life considering both eyes: results from the German population-based Gutenberg Health Study (GHS)
Source: Health Qual Life Outcomes. 2019 Jun 6;17:98. doi: 10.1186/s12955-019-1158-1 (PMC6554962; doi:10.1186/s12955-019-1158-1)
Supplement: Supplementary file 4 — Table S3a. Linear regression estimates of the influence of both the better-seeing and worse-seeing eyes on the NEI VFQ-25 visual functioning scale score in the German population-based Gutenberg Health Study (GHS), 2007–2012, restricted to participants aged younger than 65 years. (PDF 215 kb) [file 12955_2019_1158_MOESM4_ESM.pdf]

Additional file 4: Table S4: Linear regression estimates of the influence of both the better-seeing and the worse-seeing eye on the NEI VFQ-25 visual functioning scale score in the German population-based Gutenberg Health Study (GHS), 2007-2012, restricted to participants younger than 65 years (n=9015).

Category of visual impairment considering better-seeing and worse-seeing eye	Estimate (CI)	p value
**BE no VI, WE mild VI**	-6.31 (-7.58; -5.05)	< 0.0001
**BE no VI, WE moderate/ severe VI**	-8.23 (-9.41; -7.06)	< 0.0001
**BE mild VI & WE mild VI**	-21.8 (-27.2; -16.4)	< 0.0001
**BE mild VI & WE moderate/ severe VI**	-34.4 (-39.4; -29.4)	< 0.0001
**BE moderate/ severe VI, WE moderate/ severe VI**	-26.8 (-31.7; -22.0)	< 0.0001

BE: better-seeing eye; WE: worse-seeing eye; VI: vision impairment; no VI: <0.32 logMAR, mild VI: 0.32-0.5 logMAR; moderate/severe VI: >0.5 logMAR; all models adjusted for age, sex and socioeconomic status, using both eyes with no VI as reference.
