# Supplementary material for: Vision-related quality of life considering both eyes: results from the German population-based Gutenberg Health Study (GHS)
Source: Health Qual Life Outcomes. 2019 Jun 6;17:98. doi: 10.1186/s12955-019-1158-1 (PMC6554962; doi:10.1186/s12955-019-1158-1)
Supplement: Supplementary file 5 — Table S3b. Linear regression estimates of the influence of both the better-seeing and worse-seeing eyes on the NEI VFQ-25 visual functioning scale score in the German population-based Gutenberg Health Study (GHS), 2007–2012, restricted to participants aged 65 years and older. (PDF 215 kb) [file 12955_2019_1158_MOESM5_ESM.pdf]

Additional file 5: Table S5: Linear regression estimates of the influence of both the better-seeing and the worse-seeing eye on the NEI VFQ-25 visual functioning scale score in the German population-based Gutenberg Health Study (GHS), 2007-2012, restricted to participants 65 years and older (n=2874).

**Category of visual impairment considering better-seeing and worse-seeing eye**	**Estimate (CI)**	**p value**
**BE no VI, WE mild VI**	-4.06 (-5.56; -2.55)	< 0.0001
**BE no VI, WE moderate/ severe VI**	-10.7 (-12.5; -8.83)	< 0.0001
**BE mild VI & WE mild VI**	-10.8 (-15.3; -6.30)	< 0.0001
**BE mild VI & WE moderate/ severe VI**	-17.6 (-22.2; -13.0)	< 0.0001
**BE moderate/ severe VI, WE moderate/ severe VI**	-21.4 (-27.9; -14.9)	< 0.0001

BE: better-seeing eye; WE: worse-seeing eye; VI: vision impairment; no VI: <0.32 logMAR, mild VI: 0.32-0.5 logMAR; moderate/severe VI: >0.5 logMAR; all models adjusted for age, sex and socioeconomic status, using both eyes with no VI as reference.
